# Biofluorescent sexual dimorphism revealed in a southern Appalachian endemic salamander, *Plethodon metcalfi*

**DOI:** 10.1038/s41598-023-29051-8

**Published:** 2023-03-03

**Authors:** Jonathan L. Cox, Benjamin M. Fitzpatrick

**Affiliations:** 1 National Park Service, Twin Creeks Science and Education Center, Great Smoky Mountains National Park, 1316 Cherokee Orchard Rd., Gatlinburg, TN 37738 USA; 2 Department of Ecology and Evolutionary Biology, University of Tennessee, Knoxville, 596 Dabney Hall, 1416 Circle Dr, Knoxville, TN 37996 USA

**Keywords:** Herpetology, Fluorescence spectroscopy

## Abstract

Biofluorescence occurs when a living organism absorbs high energy light and reemits it at longer wavelengths. Many species within clades of vertebrates are known to fluoresce including mammals, reptiles, birds, and fish. Most, if not all, amphibians exhibit biofluorescence when exposed to either blue (440–460 nm) or ultra-violet (360–380 nm) wavelengths of light. Salamanders (Lissamphibia: Caudata) appear to consistently fluoresce in green wavelengths (520–560 nm) when excited by blue light. Biofluorescence is theorized to have many ecological functions including mate signaling, camouflage, and mimicry. Despite the discovery of their biofluorescence, its role in salamander ecology and behavior remains unresolved. In this study we present the first case of biofluorescent sexual dimorphism within Amphibia and the first documentation of the biofluorescent pattern of a salamander within the *Plethodon jordani* species complex. This sexually dimorphic trait was discovered in the southern Appalachian endemic species, Southern Gray-Cheeked Salamander (*Plethodon metcalfi*, Brimley in Proc Biol Soc Wash 25:135–140, 1912), and may extend into other species within the *Plethodon jordani* and *Plethodon glutinosus* species complexes. We propose that this sexually dimorphic trait could be related to fluorescence of ventral modified granular glands used in plethodontid chemosensory communication.

## Introduction

Biofluorescence occurs when light is absorbed and reemitted at a longer wavelength by a living organism often resulting in a magnificent glow radiating hues of blue, green, and red light^1^. This natural phenomenon has been documented with many proposed functions including sexual selection^2,3^, visual communication, camouflage, and mimicry^4^. Despite its many functions, biofluorescence has only been documented in a handful of vertebrates, including species of fish^5^, mammals^4^, reptiles^6,7^, and birds^2,3^ but recently was discovered in fourteen families of amphibians including eight families of salamanders (Lissamphibia: Caudata)^8–10^. Although most terrestrial biofluorescent organisms fluoresce under ultra-violet light, salamanders appear to mostly fluoresce when exposed to blue light (440–460 nm)^8^. This trait is similar to that of marine organisms and may be related to the relative abundance of blue wavelengths of light in aquatic^11^ and forested ecosystems^12^ or an evolutionary relict from amphibian’s marine ancestors. Lamb and Davis found that twenty-one species of salamander, across eight families, fluoresced in green wavelengths when exposed to this blue excitation light. The mechanism behind their fluorescence is poorly understood but could be related to pigments in cutaneous chromatophores such as pterin and carotenoids or reflective structures containing guanine^8^. The treefrog species, *Hypsiboas punctatus*, appears to primarily fluoresce from the presence of a molecule (Hyloin-L1; C_22_H_31_NO_4_) found within its subcutaneous lymph tissue^10^. Critically, the wavelengths of salamanders’ green biofluorescence are expected to be perceived by hetero-and conspecifics due to the green rods found in some salamanders’ eyes^13^. These green rods were originally thought to be an adaptation to assist with color differentiation in low light conditions^13^ but could also be related to the perception of green wavelengths of salamander biofluorescence in the terrestrial environment^8^. This could suggest that salamander biofluorescence is related to inter- or intraspecific visual communication and therefore sexual selection.

Fluorescence of sexually dimorphic traits, or biofluorescent sexual dimorphism, is an understudied phenomenon that has been documented only within a genus of chameleons (Squamata: *Calluma*) under ultra-violet excitation light^7^. Additionally, there is documented fluorescent sexual dichromatism in marine turtles, parrots, and blue tits where the morphologies are similar between sexes, but fluorescent intensity and wavelength may differ^2,3,6^. These dimorphic traits are hypothesized to be related to sexual selection and can theoretically be visualized by potential mates within their respective taxa. These discoveries suggest previously hidden means of communication between potential mates^7^. Salamander sexual dimorphism is often cryptic with many species having no obvious external sexually differentiating characteristics particularly outside of the breeding season^14^. Here we compared male and female Gray-cheeked Salamanders (*Plethodon metcalfi*) under blue excitation light and found a heretofore undiscovered pattern of biofluorescent dimorphism.

## Methods

**Figure 1:**
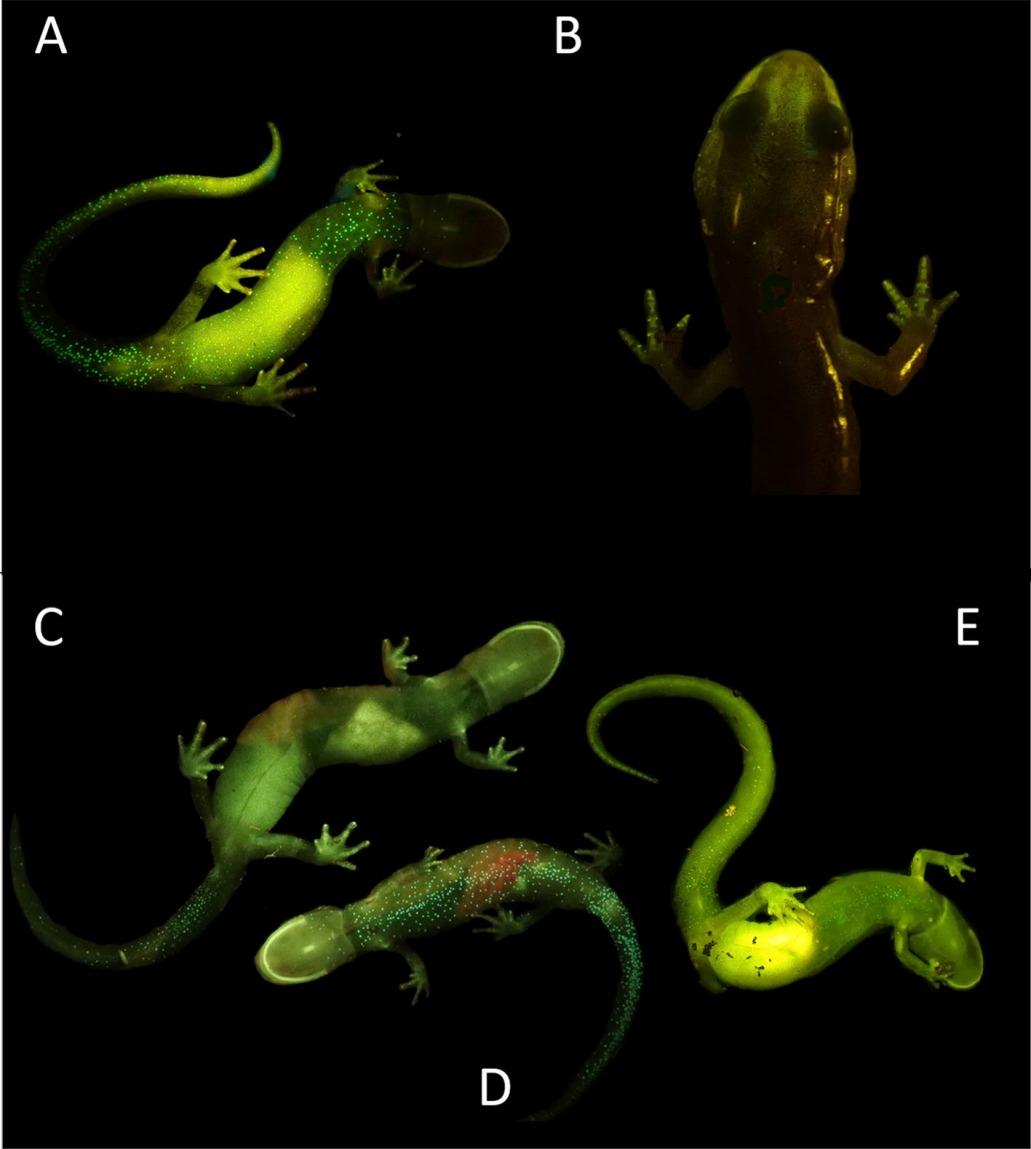
Images of the biofluorescent patterns of *P. metcalfi* filtered with a 500 nm longpass filter while exposed to blue light. (**A**) Ventral view of a male displaying the biofluorescent speckles. (**B**) Dorsal view of a male displaying the speckling down each digit with fluorescing bones. (**C**) Ventral view of a large female displaying post-cloacal speckling and fluorescent fat stores. (**D**) Male displaying the ventral trunk fluorescent speckles. (**E**) The one large female displaying less intense full ventral trunk speckling.

Handling, imaging, and spectrometer analyses of animals in this study were permitted by and conducted in accordance with all stakeholders involved including the United States National Park Service (Permit #: GRSM-2022-SCI-2173), the University of Tennessee, Knoxville Institute of Animal Care and Use Committee (Protocol #: 2899-0522), Tennessee Wildlife Resources Agency (License #: 5338), and North Carolina Wildlife Resources Commission (License #: 22-SC01508). This study was also conducted in accordance with ARRIVE guidelines^15^. As part of an ongoing study researching southern Appalachian montane salamander biofluorescence, we captured several *P. metcalfi* individuals with biofluorescent vibrant green speckling along their venters. This speckling pattern varies in density and intensity but begins just posterior to the gular fold and extends across the venter to the apex of the tail (Fig. 1). Occasionally, this pattern extends into the dorso-lateral region particularly around the base of the tail and the cheeks but is almost never seen dorsally. Throughout the course of this study, we have seen this pattern displayed by salamanders within the *P. jordani* and *glutinosus* complexes. Interestingly, this pattern was not consistent across every *P. metcalfi* individual that we sampled. We therefore hypothesized that this ventral biofluorescent speckling was sex-determined.

Sampling occurred within Great Smoky Mountains National Park in Haywood County, NC, June and July 2022 at ~ 5000 ft in elevation. We conducted both diurnal and nocturnal visual encounter and natural cover object surveys capturing all *P. metcalfi* encountered and placed them in clean plastic bags. Clean gloves were worn while handling each individual and equipment was sterilized with 0.7% Chlorohexidine gluconate if it came into contact with any salamander. Each individual was measured for SVL and examined for the presence of a mental gland. Every individual was then photographed dorsally and ventrally in a dark environment while exposed to blue excitation light (Nightsea Xite Flashlight, 440–460 nm) using a digital Olympus Tough TG-6 and/or a DSLR Canon EOS Rebel T8i camera with 500 nm longpass filters. Both cameras were set to factory macrophotography presets and exposure compensation was adjusted (− 1–0) to account for ambient light. Photographs were taken while the animals were in a plastic bag either in the field or lab depending on the proximity of their capture to the lab space. We used an OceanInsight FLAME-S-VIS–NIR–ES spectrometer equipped with a 600 nm UV/VIS fiber optic probe and linear variable longpass filter set to 500 nm to collect spectral data on each salamander across four anatomical regions in a dark environment while exposed to blue excitation light^5,8^. The sampled anatomical regions included the base of the tail just posterior to the cloaca ventrally and dorsally, and any two areas which exhibited the most intense biofluorescence, ventrally and dorsally. In one case, we opportunistically analyzed the spectral emissions of the mucous of a female individual.

We used the Wilson formula^16^ to calculate 95% confidence intervals (CI) using our observed probabilities of ventral speckling (*P*; n = 26). To compare the probabilities of ventral speckling between presumed adult males and females (n = 26) we ran contingency tables to test the null hypothesis that males and females are equally likely to have ventral speckling of any kind (α = 0.05). We then conducted a binomial regression to test the null hypothesis that SVL had no effect on presence of female ventral fluorescent speckling (n = 19). All analyses were conducted, and figures were created, in R Studio (v. 2022.02.3+492).

## Results

We collected 31 *P. metcalfi* salamanders across the two sampling periods. Of those 31 salamanders, 19 lacked a mental gland while 12 had a visible mental gland. Five of those 19 lacking mental glands appeared to be juveniles with SVLs < 40 mm and displayed no fluorescent speckling. We did not verify sex beyond the presence of a mental gland, therefore we know that every individual with a mental gland is male but there is a possibility of individuals without a mental gland being male or female. However, during the breeding season sexually mature males should have a mental gland therefore we refer to individuals with mental glands as males and those without as females.

**Figure 2:**
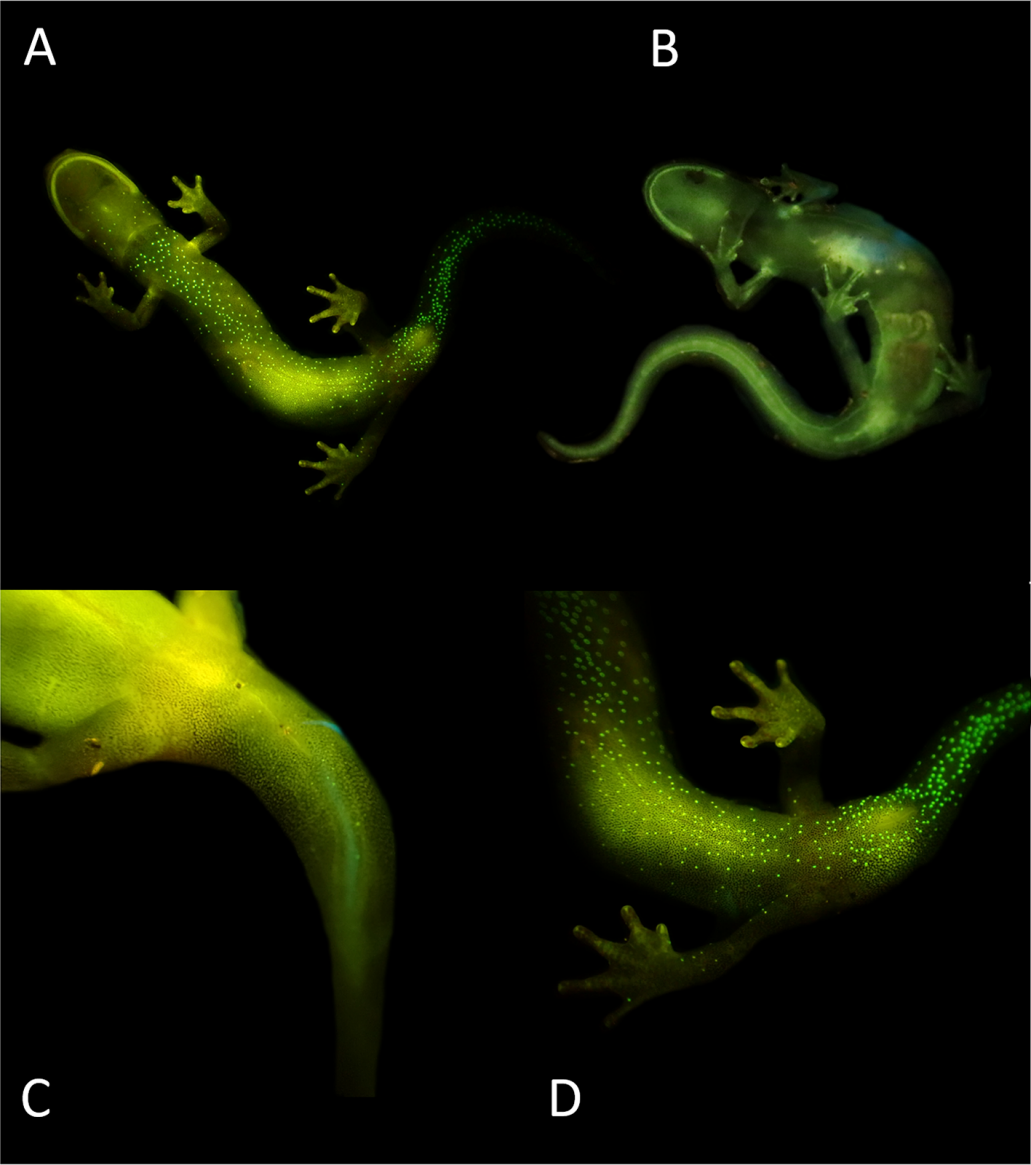
Photographs of *P. metcalfi* salamanders taken with a 500 nm longpass camera lens filter while exposed to blue excitation light. (**A**) Ventral surface of a male *P. metcalfi* displaying biofluorescent speckling. (**B**) Ventral surface of a female *P. metcalfi* lacking biofluorescent speckling. (**C**) Cloacal region of a female with minimal speckling and the (**D**) cloacal region of a male displaying biofluorescent speckling.

**Figure 3:**
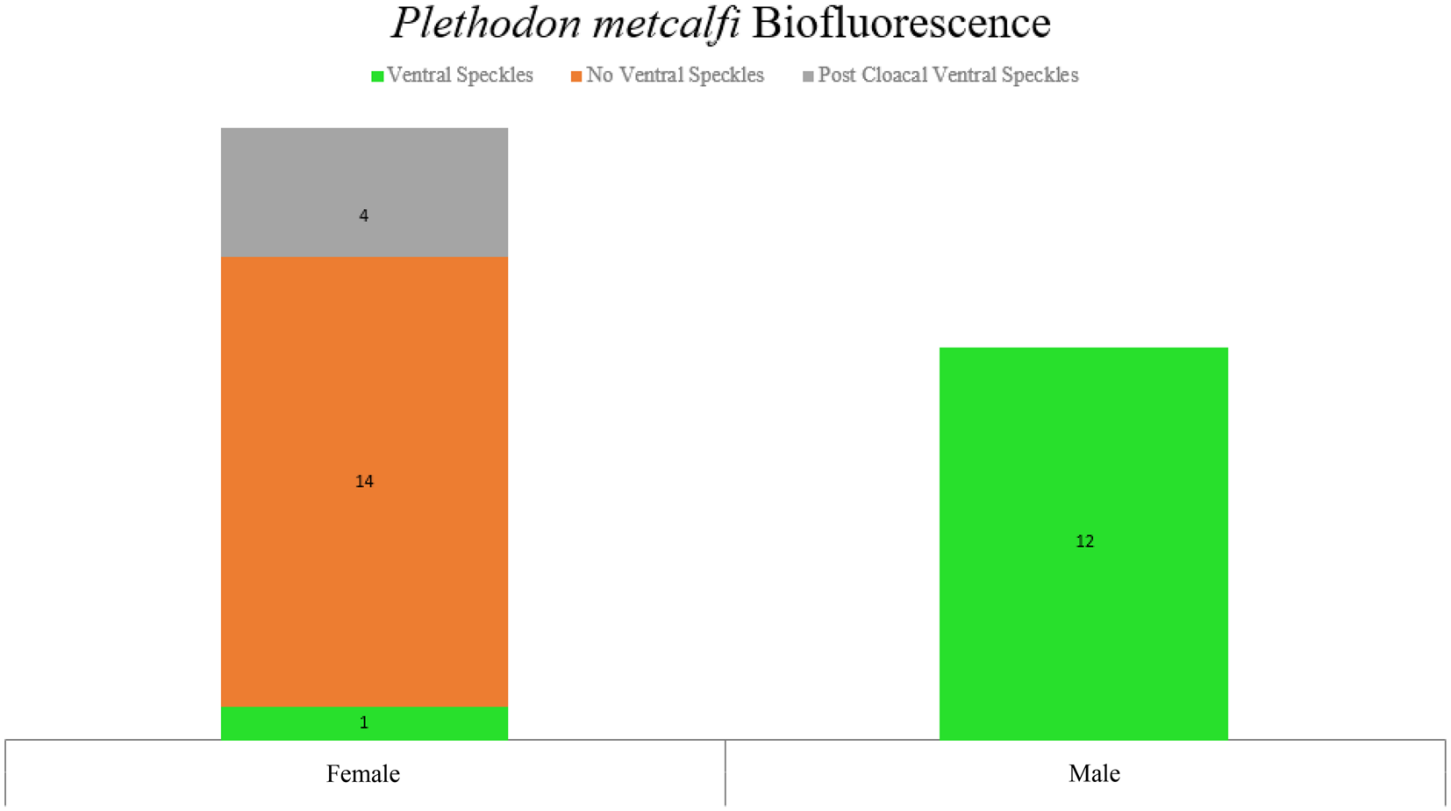
Bar graph showing the number of biofluorescent phenotypes observed between sexes, or mental gland presence.

**Figure 4:**
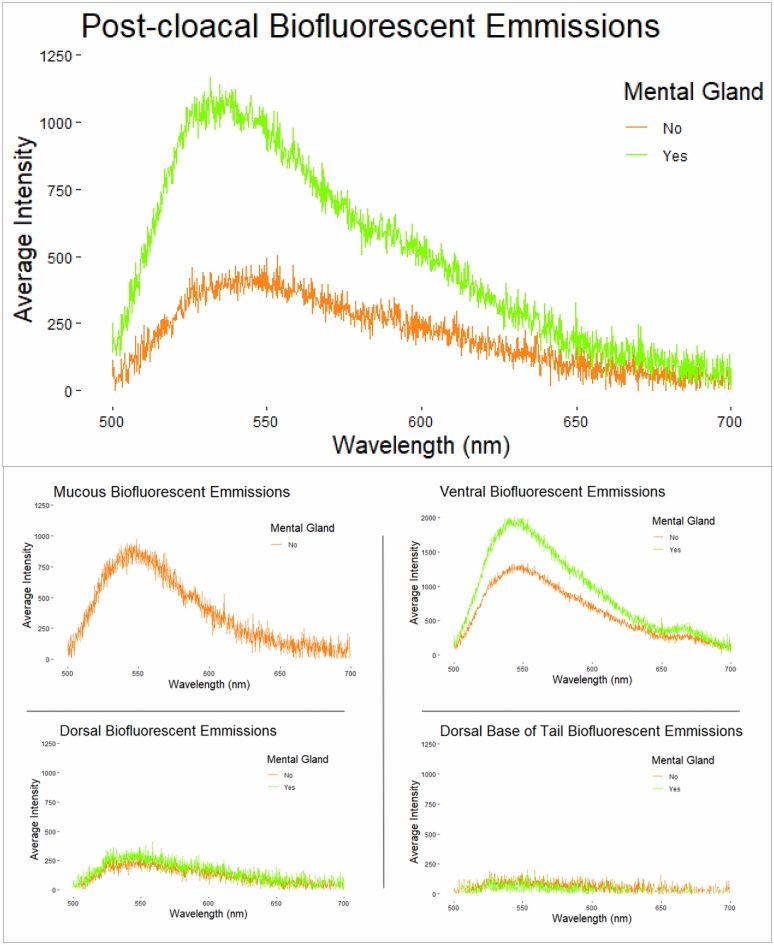
The biofluorescent spectral emissions averaged by male (green; n = 12) and presumed female (orange; n = 19) *P. metcalfi* salamanders recorded while exposed to blue light and filtered with a 500 nm longpass filter. Each graph represents a different anatomical region or mucous (n = 1) sample.

All 12 males displayed vibrant fluorescent green speckles along the ventral trunk beginning roughly posterior to the gular fold extending down to the apex of the tail (Figs. 1A,D, 2A,D). This pattern was seen extending into the dorso-lateral region particularly around the cheeks and base of the tail in 7 males, but in one case extending down the length of the trunk dorso-laterally. In 9 cases, males displayed paired speckles, of similar appearance, symmetrically down each digit of every foot visible from both the dorsal and ventral angles (Fig. 1B). Of the 14 adult females, or individuals without mental glands, one was captured with the same vibrant green speckling pattern but with less intensity (Fig. 1E). The 13 remaining individuals either entirely lacked this speckled pattern or the pattern was found with less density and only posterior to the cloaca extending down the tail (Figs. 1C, 2B,C, 3). This pattern of post-cloacal speckling was noted only in females with SVLs of µ.70 mm or greater (n = 4). Regardless of sex, every individual fluoresced from green to orange (520–650 nm) wavelengths across the entire body, including their bones, with specific anatomical regions emitting more intense light. Average peak intensities were measured at green wavelengths between 520 and 550 nm regardless of anatomical region (Fig. 4).

**Figure 5:**
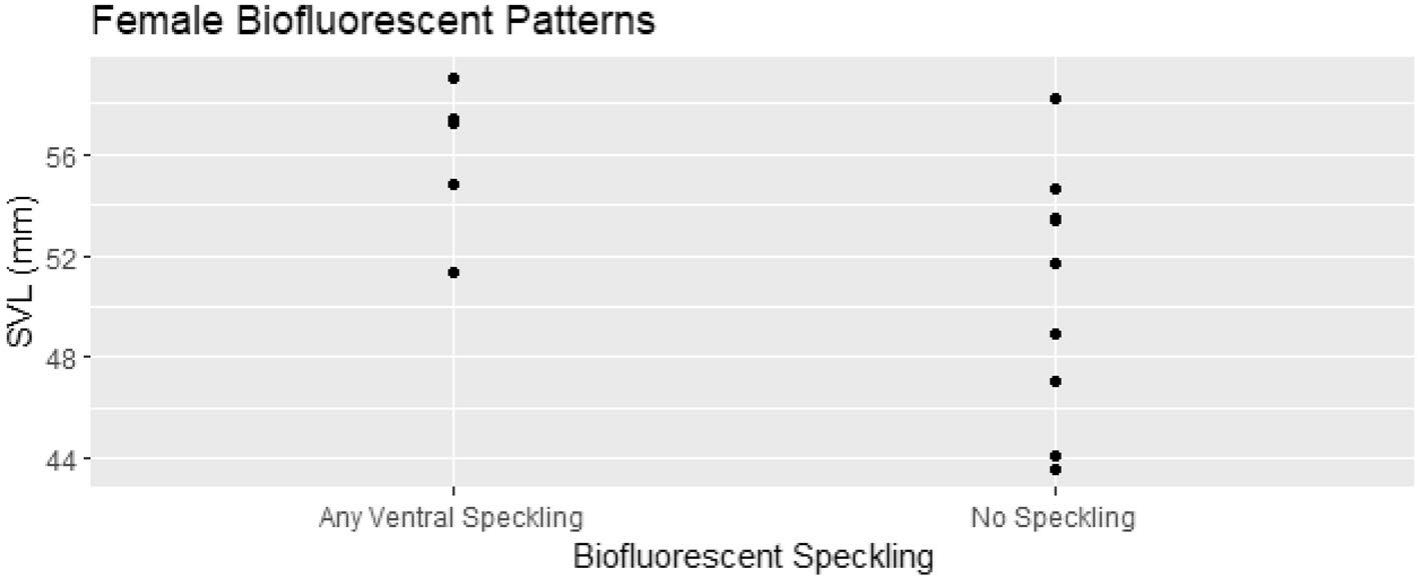
Scatter plot of female biofluorescent speckling with snout-vent length.

From the results of the Wilsons test, the upper and lower CI’s of both males and females speckling probability do not overlap (Table 1). Our contingency table results indicate that the probabilities of sex-determined speckling, either entirely or posterior to the cloaca, are significantly different (p ≤ 0.0001 & p = 0.0011 respectively; Table 1). We then ran a binomial regression followed by a type II ANOVA testing the relationship between SVL and ventral speckling for presumed females. Snout-vent length was a significant predictor of ventral speckling within female *P. metcalfi* (p = 0.026; AIC = 17.28, z = 1.68, n = 14; Fig. 5).

**Table 1:** Point estimates and 95% confidence intervals (using the Wilson method) for the prevalence of biofluorescent speckling in presumed male and female *Plethodon metcalfi*.

Mental gland	n	X	P	Lower	Upper	χ^2^ (df = 1)	p-value
Present	12	12 (full speckling)	1.0000	0.7575	1.0000	–	–
Absent	14	1 (full speckling)	0.0714	0.0127	0.3146	18.73	< 0.0001
		5 (any speckling)	0.3571	0.1634	0.6123	9.13	0.0011

Males fluoresced more intensely in almost every anatomical region that was sampled (Fig. 4). The dorsum of both males and females fluoresced in relatively low intensity but peaked within the same wavelengths. Most individuals fluoresced the most intensely on their feet, seemingly related to their digits fluorescing (Fig. 1B), and their ventral trunk within what appeared to be fat stores (Fig. 1A,E). Additionally, the mucous of one female was found to be fluorescent peaking around 550 nm in wavelength (Fig. 4).

## Discussion

*Plethodon metcalfi* salamanders are inconspicuous amphibians under ambient light. Since their first discovery, these salamanders have been described as steel gray to black with no distinct coloration, in contrast to some of their vibrantly and aposematically colored relatives^17^. Although we may be presenting the first recorded case of *P. metcalfi* biofluorescence, and the first documented case of biofluorescent sexual dimorphism in Lissamphibia, it should be considered that our sample size of adult salamanders is relatively small (n = 26). The sampled salamanders fluoresce across their entire body with more vibrant patterns displayed on their ventral side seemingly dependent on their sex and size. These salamanders fluoresce when exposed to blue excitation light within the green to orange visible range (520–650 nm). Their peak biofluorescent intensities occur between 520 and 550 nm wavelengths regardless of their sex or where we measured their biofluorescent emissions. *Plethodon metcalfi* males exhibited vibrant biofluorescent green speckling across the entirety of their ventral trunk beginning just anterior to the gular fold and extending to the apex of the tail. In contrast, most presumed females exhibited no vibrant biofluorescent speckling whatsoever except 5 larger individuals. These larger females displayed similar speckling to the males but beginning just posterior to the vent and extending down to the apex of the tail. In one case, a presumed female exhibited the exact same speckling pattern as a male. These larger biofluorescent females could simply be males lacking a prominent a mental gland or are indeed females displaying their own unique biofluorescent pattern.

Salamander biofluorescence is a novel natural history and scientific phenomenon^8,9^. Their biofluorescent patterns are hypothesized to be derived from pigments found in cutaneous membranes^8^ but the recently discovered *Hypsiboas punctatus* biofluorescence was derived from a molecule (Hyloin-L1) found in its subcutaneous lymph tissue^10^. The discovery of biofluorescent sexual dimorphism may enlighten our understanding of how and why some salamanders fluoresce at all. The presence of this dimorphism leads one to believe that biofluorescence plays a role in salamander communication, sexual selection, or is simply an evolutionary relict dimorphically expressed due to the physical or chemical composition of the trait. Although, other amphibian’s biofluorescence has been documented as more intense than ambient reflectance^10^ we suspect that *P. metcalfi*’s biofluorescent sexual dimorphism may be unrelated to visual communication due to their natural history. *Plethodon metcalfi* is a fully terrestrial species which undergoes direct development meaning their ventral side is always parallel or in contact with the forest floor. Therefore, it seems unlikely that a female would have the opportunity to visualize the biofluorescence of a male’s ventral trunk unlike a semi-aquatic or aquatic species. However, there is an exception: during *Plethodon* courtship, males will make contact on a female’s integument with their mental gland and perform what’s known as a “foot dance”^18,19^. Organ^18^ describes the “foot dance” as an alternation between raising and lowering all of the limbs with raising and lowering only the fore or hindlimbs. During this behavior it is possible that a female could visualize the male’s symmetrical fluorescent speckles that run down each digit (Fig. 1B).

Considering the anatomical location and this species natural history, we speculate that *P. metcalfi*’s biofluorescent speckling is derived from skin glands found on the ventral trunk of male, and large female, *P. metcalfi*. These glands or pores can be seen under white light in preserved specimens although they no longer fluoresce under blue light after preservation (Jonathan L. Cox, personal observation). This loss of fluorescence in wet preservation could suggest that the fluorescence is caused by a glandular pigment or the chemical composition of the gland secretions and not by the gland structure. While *Plethodon* salamanders have many types of skin glands along their ventral trunk, modified granular glands (MGGs) have been found to be sexually dimorphic. These dimorphic skin glands are referred to as sexually dimorphic skin glands (SDSGs) and are related to courtship and chemical communication^20–23^. Male *Plethodon* salamanders often use scent marking to attract females through SDSGs particularly via the mental gland and caudal courtship glands^20,22^. In *P. metcalfi*, neither of these glands, or where they occur on the body, fluoresced with comparable intensity to the male ventral speckling. Therefore, we suspect that the biofluorescent speckling could be derived from MGGs, or possibly what Simons et al. described as serous acini glands, which are used in territorial or advertisement scent marking seen in other *Plethodon* salamanders^24–29^. Scent marking behaviors observed within *P. cinereus* utilize these ventral MGGs or acini glands to produce pheromones that appear to claim territory or alert allospecifics and conspecifics to their presence^24–26,28^. Although both males and females display these behaviors, the MGG morphology, pheromones produced, and response behaviors likely differ^22,24,26^. Therefore, it seems possible that if there is sexual dimorphism in plethodontid scent marking MGG size^22^, pheromones, and behavioral response^26^ then these glands could be the source of biofluorescent sexual dimorphism. This could also explain the presence of the occasional biofluorescent speckling observed in large female *P. metcalfi*. In other species of *Plethodon*, males are known to select for larger females possibly due to an association between larger SVL and higher fecundity^30^. Jaworski et al.^30^ also noted that male Plethodon cinereus salamanders utilize a higher frequency of ‘nose tap’ behavior towards larger females. This likely indicates that males utilize the chemical cues of larger females more often. If these biofluorescent speckles are indeed related to scent marking MGGs, large females could be expressing them in order to signal to dominant males.

Further research should be conducted to confirm our speculation of MGG fluorescence by illuminating ventral tissue cross sections with blue light during microscopy. Additionally, it seems pertinent to view *P. metcalfi* courtship behaviors while exposed to blue excitation light to confirm windows of opportunity that females have to perceive their biofluorescence during the “foot dance”. The intensity and wavelength of male’s fluorescence could be an avenue of sexual selection when visualized during this courtship behavior. The ventral trunks and limbs of other related Plethodon salamanders should also be viewed under blue excited light, particularly those which may not utilize the “foot dance” as often as their congeners. Pierson et al.^19^ noted that male *Plethodon yonahlossee* were only documented performing the “foot dance” once throughout their study, and at an unusual stage of courtship. By analyzing male *P. yonahlossee* salamanders under blue excited light we could shed light on the utility of biofluorescent limbs in the “foot dance” behavior. Furthermore, a complete investigation of the biofluorescent patterns of the *P. jordani* and *P. glutinosus* species’ complexes should be conducted. Our observed *P. metcalfi* biofluorescent sexual dimorphism may be a common trait among these related clades. Regardless of the biofluorescent ecological function, blue excited light may now prove useful as a method of sexual identification among *P. metcalfi* salamanders outside of the breeding of the season. Although, it should not be considered foolproof until further research is conducted confirming sexes, and breeding phenology, alongside blue excited light fluorescence.

## Data Availability

The datasets used and/or analyzed during the current study is available from the corresponding author on reasonable request.
